# Computational modelling of patient specific spring assisted lambdoid craniosynostosis correction

**DOI:** 10.1038/s41598-020-75747-6

**Published:** 2020-10-29

**Authors:** Selim Bozkurt, Alessandro Borghi, Lara S. van de Lande, N. U. Owase Jeelani, David J. Dunaway, Silvia Schievano

**Affiliations:** 1 Institute of Cardiovascular Science, University College London, London, UK; 2 University College London, Great Ormond Street Institute of Child Health, London, UK; 3 Craniofacial Unit, Great Ormond Street Hospital for Children, London, UK

**Keywords:** Bone development, Bone remodelling, Computational science

## Abstract

Lambdoid craniosynostosis (LC) is a rare non-syndromic craniosynostosis characterised by fusion of the lambdoid sutures at the back of the head. Surgical correction including the spring assisted cranioplasty is the only option to correct the asymmetry at the skull in LC. However, the aesthetic outcome from spring assisted cranioplasty may remain suboptimal. The aim of this study is to develop a parametric finite element (FE) model of the LC skulls that could be used in the future to optimise spring surgery. The skull geometries from three different LC patients who underwent spring correction were reconstructed from the pre-operative computed tomography (CT) in Simpleware ScanIP. Initially, the skull growth between the pre-operative CT imaging and surgical intervention was simulated using MSC Marc. The osteotomies and spring implantation were performed to simulate the skull expansion due to the spring forces and skull growth between surgery and post-operative CT imaging in MSC Marc. Surface deviation between the FE models and post-operative skull models reconstructed from CT images changed between ± 5 mm over the skull geometries. Replicating spring assisted cranioplasty in LC patients allow to tune the parameters for surgical planning, which may help to improve outcomes in LC surgeries in the future.

## Introduction

Lambdoid craniosynostosis (LC) is a rare type of craniosynostosis where the lambdoid sutures are fused^1–3^. It can take place in bilateral or unilateral form or may even exist along with other types of cranial deformities^4^, and is associated with herniated cerebellar tonsils^5^. Fused lambdoid sutures in an LC skull cause shape asymmetry in the back of the skull, which may in turn result in further problems, such as raised intracranial pressure or torticollis because of developing positioning preference and shortening of the ipsilateral sternocleidomastoid muscle^6,7^. Surgical intervention is the only treatment to expand the cranial vault in LC and thus correct the asymmetry in the skull^8^. Different surgical approaches such as endoscopic strip suturectomy, bone flap remodelling or switching, distraction osteogenesis or spring assisted correction may be adopted to correct the deformity^9–14^, usually before 12 months of age^15^. Nonetheless, aesthetic outcomes of the surgical correction in LC generally remain suboptimal, with persisting asymmetry at the cranial base and posterior cranial vault^16^.

Springs were first used at Sahlgrenska University Hospital to correct cranial vault postoperatively^17,18^. Spring assisted cranioplasty is performed mainly to correct scaphocephaly, the most common craniosynostosis type^19,20^, but also for patients with a brachycephalic head shape due to (bi) coronal craniosynostosis by performing a posterior vault expansion. Modifications have been introduced for head shape correction in anterior plagiocephaly and metopic synostosis^21–23^. Spring assisted correction of lambdoid craniosynostosis has been reported, where it was part of a multi-sutural deformity^24,25^. The surgery requires insertion of spring distractors in the skull after osteotomies are performed to release the fused sutures; the springs, initially compressed, start opening resulting in an expansion force to the skull perpendicular to the osteomised cranial bone. Although spring assisted cranioplasty requires a second operation to remove the devices^26^, it has the advantages of providing increase in volume and circumference of the cranium, whilst being minimally invasive, thus reducing procedural morbidity and requiring relatively short operative time and hospital stay^23,26,27^.

Understanding the 3D asymmetry in spring assisted LC correction or simulating the treatment using a patient-specific skull model may help improve the outcome of this procedure. Finite element (FE) analyses have already been utilised to simulate correction of cranial deformities. For instance, Wolanski et al. focused on sagittal and metopic craniosynostosis correction^28^; Borghi et al. simulated spring assisted correction of sagittal craniosynostosis in patient-specific models^29^; Malde et al. developed a patient-specific FE model of sagittal craniosynostosis to predict calvarial morphology^30^; and Bozkurt et al. evaluated potential correction methods for unicoronal craniosynostosis using a patient-specific FE skull model^31^. Numerical studies aiming to simulate skull correction focus on common craniosynostosis types such as sagittal, unicoronal or metopic synostosis. Therefore, simulation of isolated LC correction remains to study.

The aim of this study is to simulate spring assisted correction in isolated LC patients using patient-specific skull models via parametric FE analyses which can provide useful insights to improve the outcome of spring assisted cranioplasty.

## Methods

Data was analysed in accordance with the guidelines laid out in the Declaration of Helsinki. Ethical approval was obtained for the collection, storage and analysis of the tissue samples (UK REC 09/H0722/28) and use of image data for research purposes (UK REC 15/LO/0386) from the Joint Research and Development Office of Great Ormond Street Hospital for Children. All parents/guardians gave written informed consent to participate in this study.

**Figure 1:**
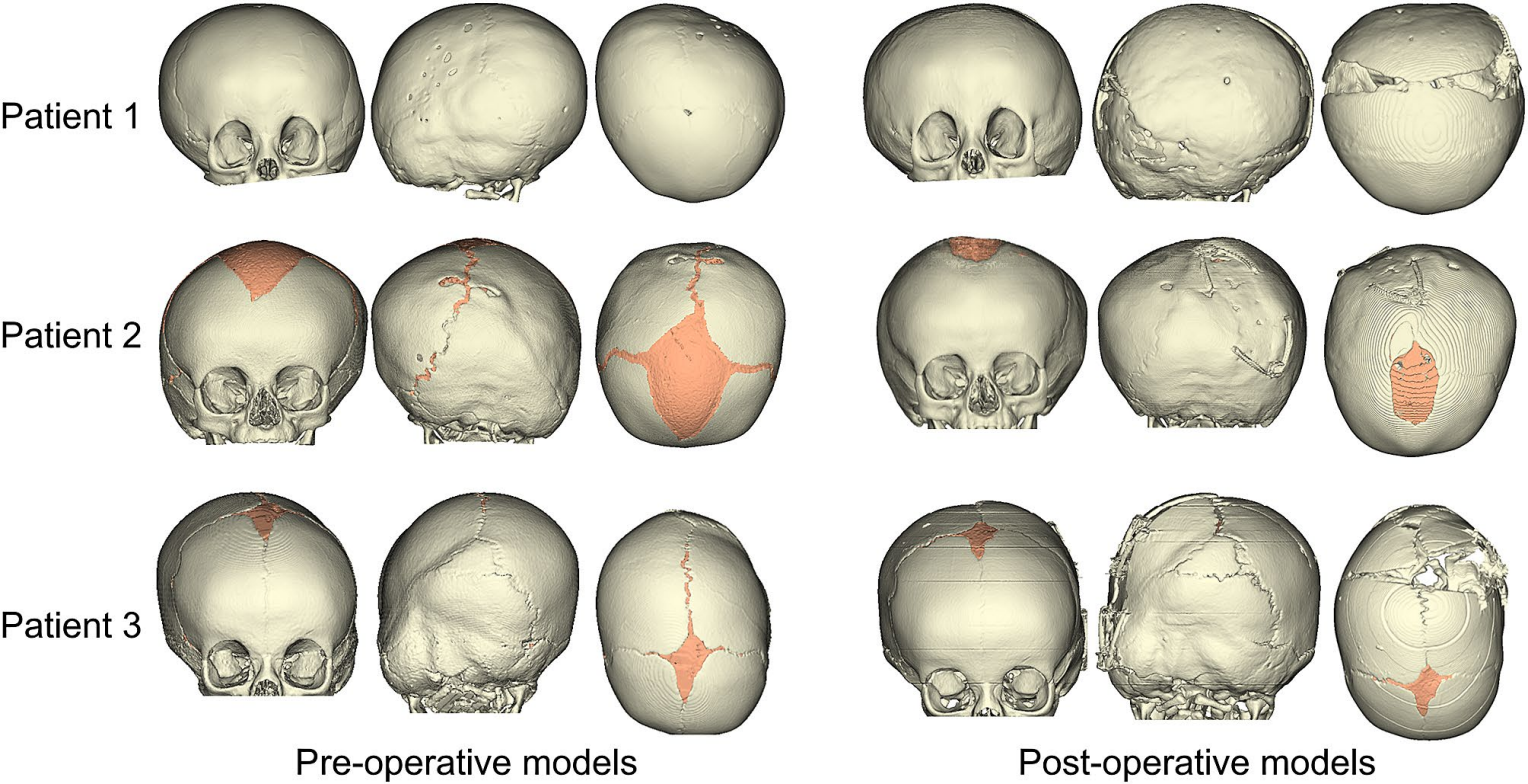
Patient-specific pre and post-operative skull models reconstructed from the CT images.

Three LC patients who underwent spring assisted surgery for abnormal skull shape at our Craniofacial Unit with pre- and post-operative computed tomography (CT) images were selected for this study. The patients were 196 (patient 1), 134 (patient 2) and 104 (patient 3) days old at time of pre-operative CT scan imaging; they underwent surgery at 242, 196 and 199 days of age; and the post-operative scans were acquired at the age of 317, 420 and 210 days, respectively. Patient specific skull models were reconstructed from the CT images in Simpleware ScanIP, including the bone of the calvarium to the maxilla and the suture structures. The pre- and post-operative patient specific reconstructions are shown in Fig. 1.

Structural 3D tetrahedral elements were used to mesh and create the skull FE models (354,359, 672,269 and 574,283 in each model, respectively). Materials were modeled as linear elastic with Poisson ratio (ν) equal to 0.49 for the sutures and 0.22 for the bony parts, whilst the Elastic modulus (E) was selected according to the patient age. Validated parametric FE models showed that average Elastic modulus of skull bone in 0–9 month old children is around 157 MPa and for sutures 8.3 MPa^32,33^. However, these values change significantly with age^34^. Therefore, Elastic modulus of the bony part was selected as 157 MPa for the first model and 85 MPa for the other two models considering the age of the patients at the intra-operative time. Elastic modulus of the sutures was 8.3 MPa for all patients. Fixed nodal displacement and rotation boundary conditions were applied at the base of the models.

Exponential increase in skull size results in a high growth rate in intracranial volume (ICV) during the first 12 months of life and in a significantly reduced growth rate after 5 years of age^35^. Therefore, skull growth between the pre-operative CT imaging and surgical intervention time was simulated in the FE package MSC Marc before performing the osteotomies on the skull models. ICV was used as the parameter representing skull size as described in^36^. ICV at time of surgery was estimated utilising an empirical model^37^ which predicts the skull growth until 18 years of age as

1 $$ {\text{ICV}}_{{\text{h}}} \;({\text{t}}) = 157.9\;{\ln}({\text{t}}) + 104.1. $$

**Figure 2:**
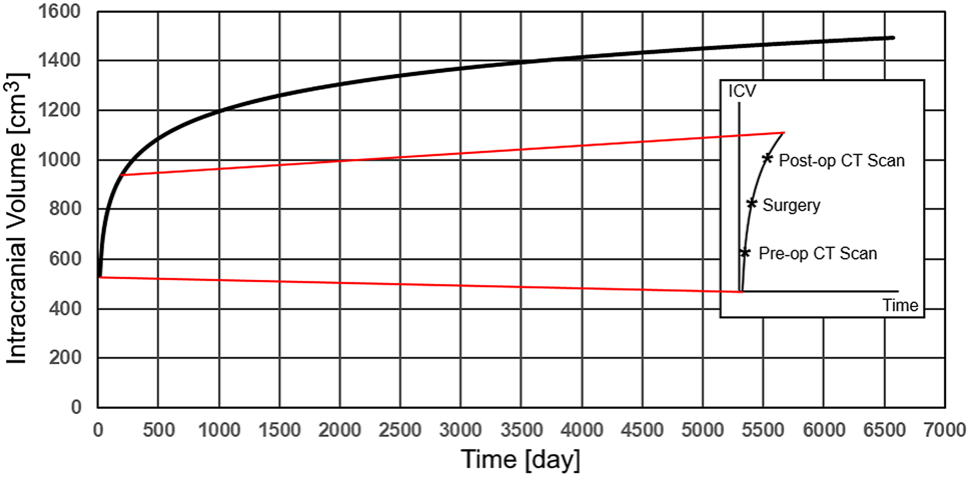
Intracranial volume (ICV) simulated by the model in Eq. (1) and region of interest on the growth curve with representative pre-operative, surgical intervention and post-operative times.

Here, *ICV*_*h*_ represents the ICV in healthy subjects and *t* represents time. Surgical intervention in LC skulls is generally performed before 12 months of age, as in the analysed patients^15^. Therefore, a small portion of the curve covering the times between pre-operative CT imaging and surgical intervention was used to predict skull growth in the simulations, as shown in Fig. 2.

ICV growth in the LC patients was assumed proportional to ICV growth of healthy subjects. A coefficient (*k*) describing the ratio between ICV in LC patients (*ICV*_*LC,pre*_) and ICV in healthy subjects (*ICV*_*LC,pre*_) was defined as:

2 $$ {\text{k}}\, = \frac{{{\text{ICV}}_{{\text{LC,pre}}} ({\text{t}})}}{{{\text{ICV}}_{{\text{h,pre}}} ({\text{t}})\,}}. $$

Thus, intra-operative ICV (*ICV*_*LC,intra*_) at time of surgery in the LC skull models was estimated for each patient as.

3 $$ {\text{ICV}}_{{\text{LC,intra}}} ({\text{t}})\, = {\text{k}} \times {\text{ICV}}_{{\text{h,intra}}} ({\text{t}}). $$

Skull growth between the pre-operative CT scan and surgical intervention was implemented for each model in MSC Marc using a similar method to that proposed by Libby et al.^38^ who approximated skull growth to a thermal expansion as

4 $$ {\text{V}}_{{\text{LC,intra}}} - {\text{V}}_{{\text{LC,pre}}} = {\text{V}}_{{\text{LC,pre}}} \times \alpha \times \Delta {\text{T}}. $$

Here, V represents size of the bony and soft tissue parts of the skull, α is the expansion coefficient and ΔT is the temperature difference.

The ICV was measured from the pre-operative CT reconstructions by selecting the internal surface of the cranial vault in Simpleware ScanIP.

**Figure 3:**
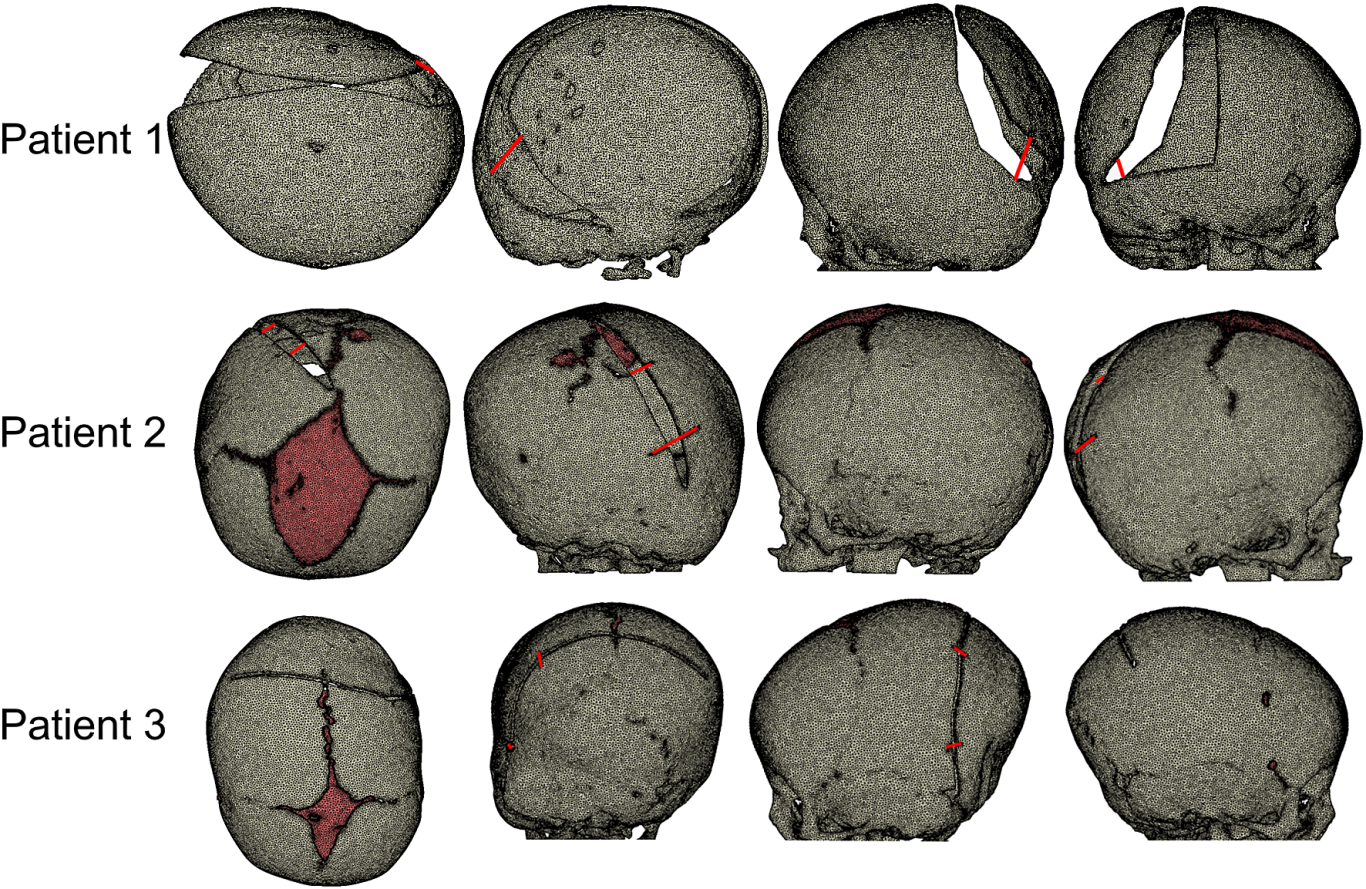
The FE models simulating spring assisted cranial expansion with osteotomies.

The osteotomies on the skulls performed at the time of surgery were replicated on the skull geometries after reaching the intra-operative estimated ICV by following the traces remaining visible from the surgery on the post-operative skull models in Simpleware ScanIP. The skull geometries with osteotomies were re-meshed using structural 3D tetrahedral elements (450,744, 782,668 and 767,282 for patient 1, 2 and 3, respectively). Spring implantation was simulated using spring/dashpot link elements in MSC Marc, by specifying spring stiffness (1.2 mm wire diameter springs were used in Patient 1, and 1.4 mm wire diameter in Patient 2 and Patient 3) and initial force in a compressed spring according to the characteristics reported in^39^. The skull growth between surgical intervention and post-operative CT scan was simulated using the methods described in Eq. (4). The temperature difference (ΔT) was 100 K in all pre and post-operative FE models. The FE models with osteotomies and springs are shown in Fig. 3.

Surface deviation between the expanded FE skull models and post-operative CT skull reconstructions was assessed in Simpleware ScanIP, after volume registration achieved using landmarks on the anterior nasal spine and frontozygomatic sutures, not affected by the surgery^40^. Simulations were performed iteratively by tuning the expansion coefficients (α) until the average surface distance was within ± 1 mm of the post-operative CT reconstructions for all cases and the average negative and positive surface deviations were between − 1 mm and + 1 mm respectively for the entire skull in each model.

## Results

**Figure 4:**
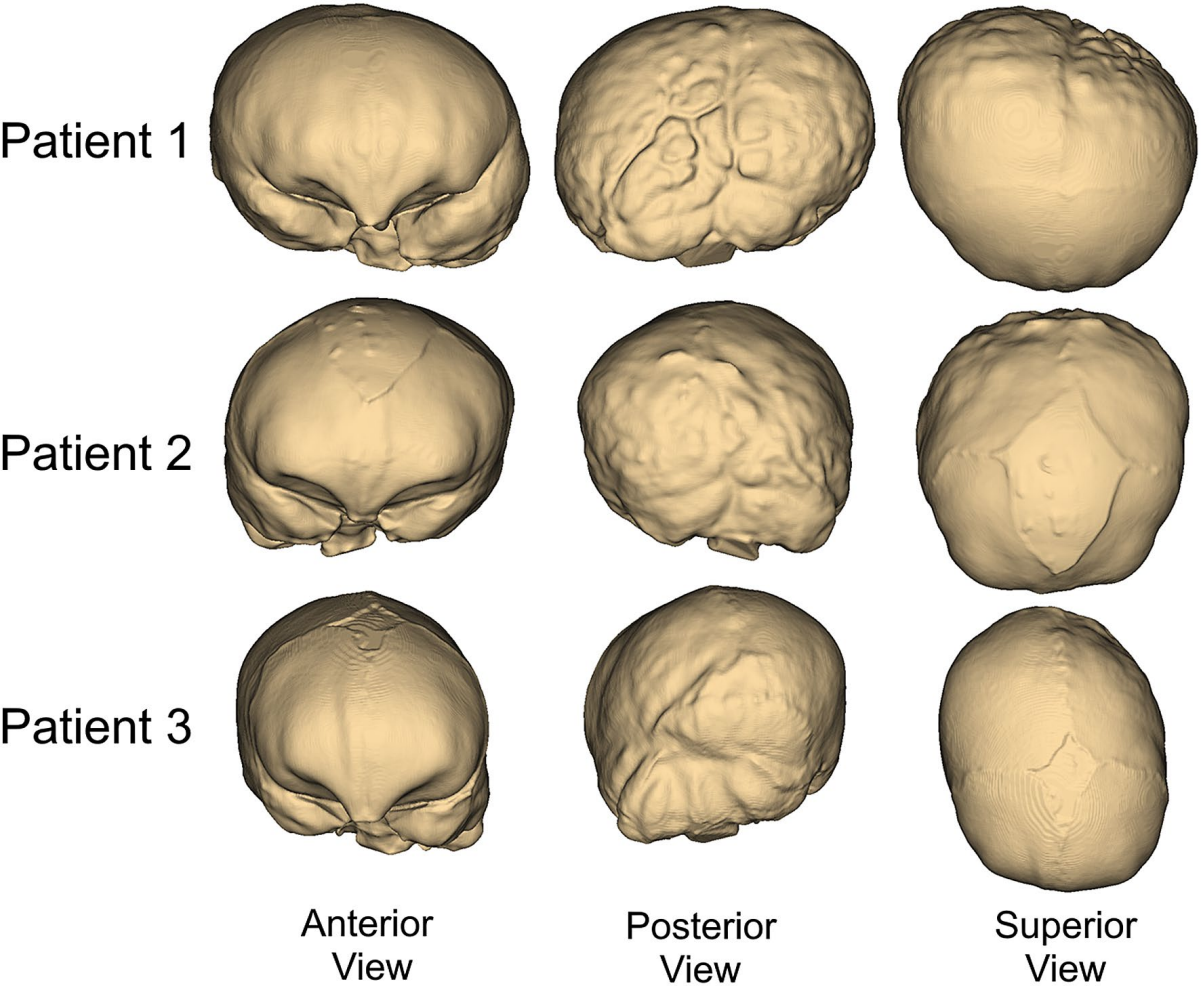
Shapes of the intracranial cavities in the patients’ skulls.

Shapes of the intracranial cavities at the time of pre-operative CT in each patient are given in Fig. 4. Premature fusion of the lambdoid suture creates flattening in the posterior skull and deformities due to LC are also noticeable in the intracranial geometries of the patients (Fig. 4).

The tuned expansion coefficients used in the thermal FE models simulating skull growth between the pre-operative CT scans and surgical intervention are given in Table 1. Pre-operative ICV measured from CT, and ICV at the time of surgery estimated from growth curve and simulated in the FE models are reported in Table 1. The FE values match well the intraoperative ICV volumes. Patient 1 had the largest pre and intra-operative ICVs among all the patients whilst a relatively small expansion coefficient was used to simulate skull growth in it. Although, Patient 2 and Patient 3 had similar pre-operative ICVs, Patient 3 had a larger intra-operative skull size.

**Table 1:** Thermal expansion coefficients pre-operative ICV, estimated and simulated ICVs in the FE models simulating skull growth between pre-operative CT scans and surgical interventions.

	Thermal expansion coefficient (K^−1^)	Pre-operative ICV from CT measurements (mL)	Estimated intra-operative ICV from growth curves (mL)	Simulated intra-operative ICV from FE models (mL)
Patient 1	0.00012	1105	1144	1145
Patient 2	0.00023	725	774	774
Patient 3	0.00039	729	818	818

**Figure 5:**
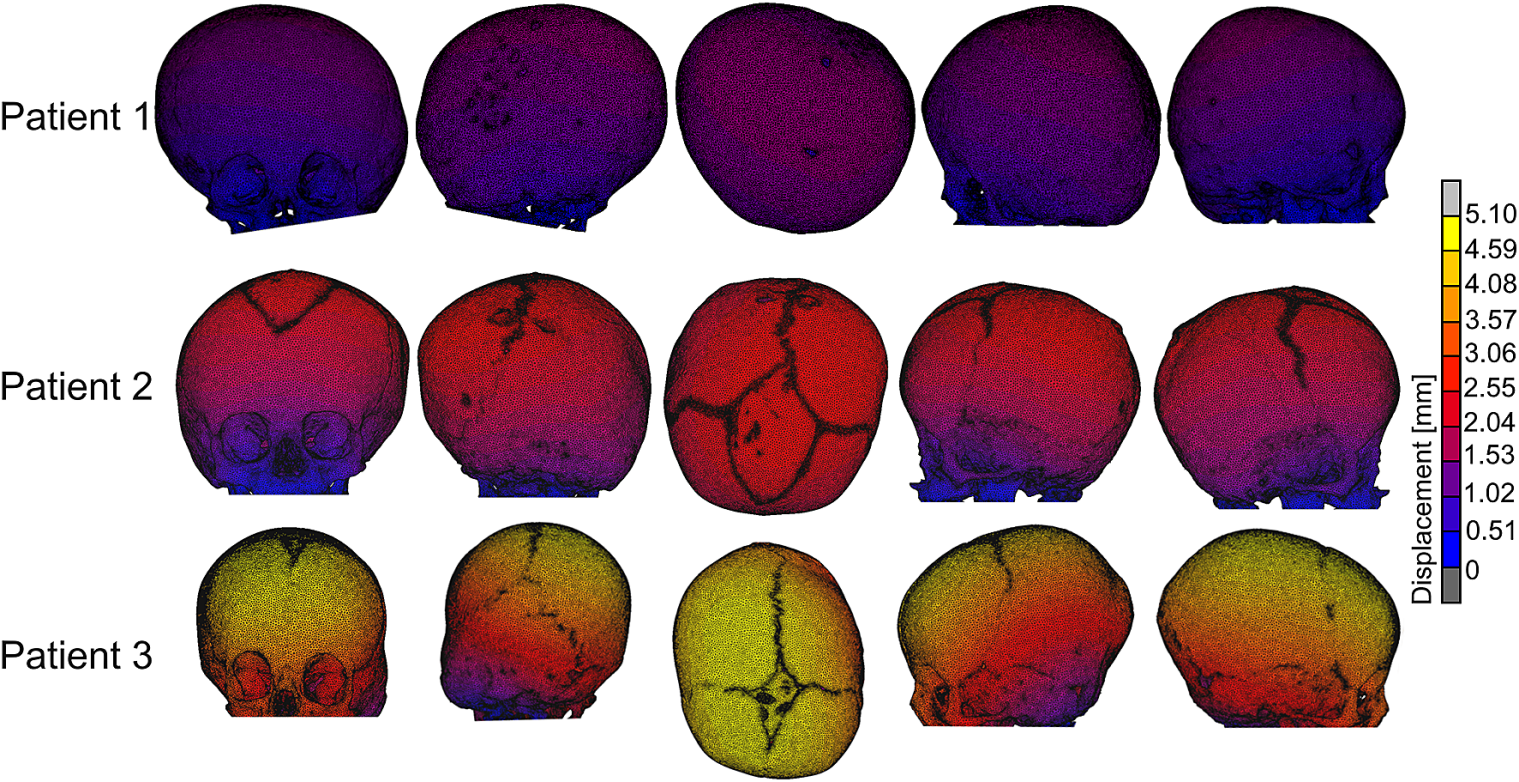
Displacement maps for the FE models simulating the skull growth between the pre-operative CT scan and surgical intervention.

Displacement maps for the FE models simulating the skull growth between the pre-operative CT scan and surgical intervention are given in Fig. 5. Maximal displacements in the skull models of Patient 1, Patient 2 and Patient 3 were 1.49 mm, 2.55 mm and 5.10 mm, respectively. Relatively high displacements are achieved in the Patient 3 skull model due to relatively young age, therefore, a higher expansion coefficient used in the simulations to achieve the estimated intra-operative ICV.

**Figure 6:**
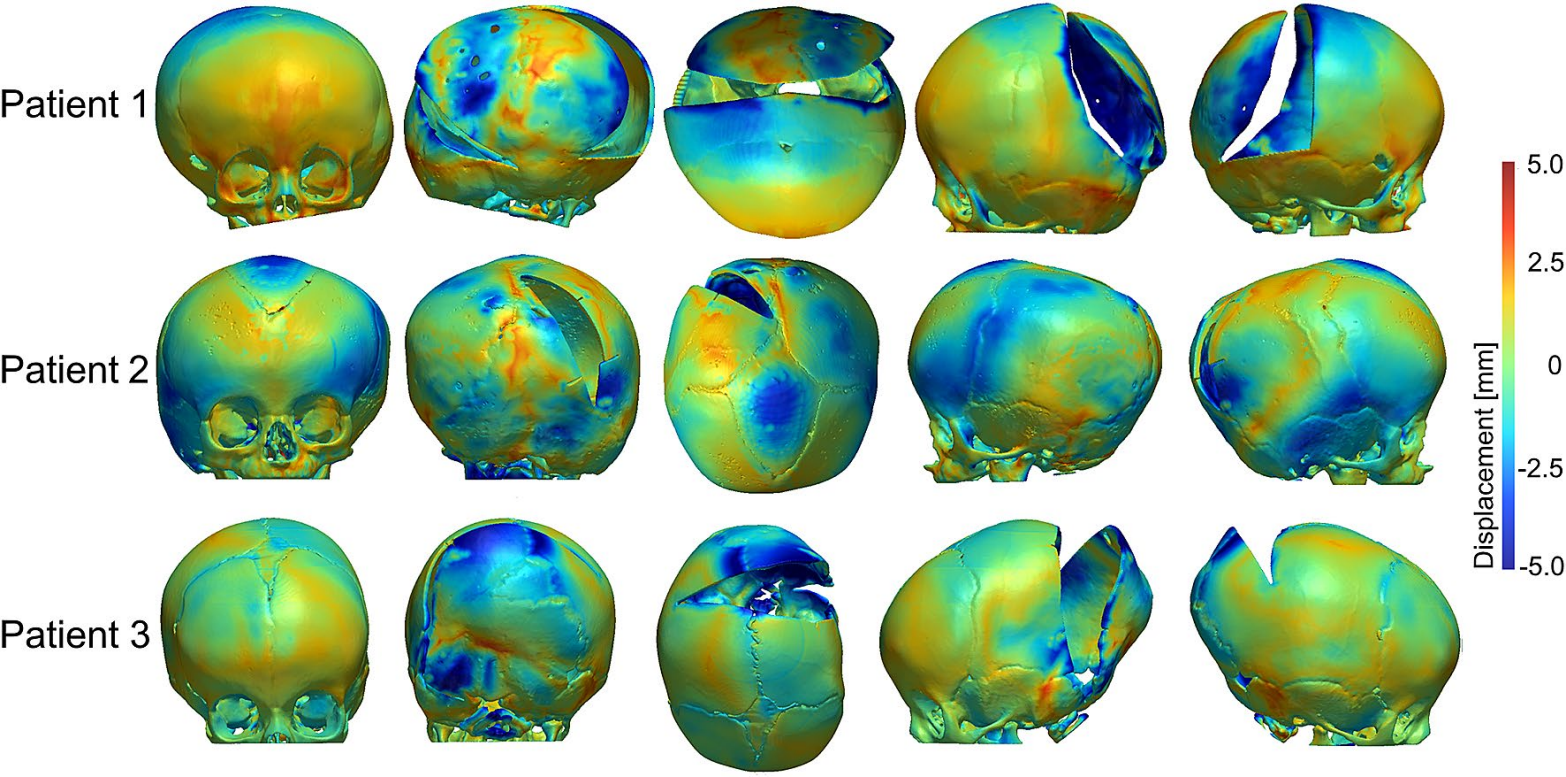
Surface deviation between the FE models and post-operative skull models reconstructed from CT images.

**Figure 7:**
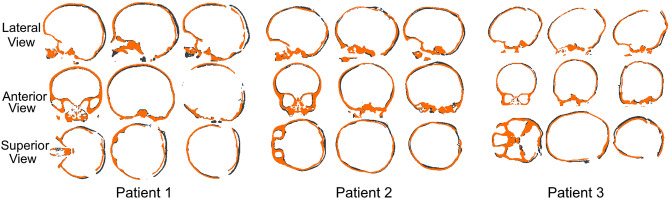
Comparison between the cross-sections of the FE models simulating spring assisted cranioplasty (orange) and post-operative skull growth and post-operative skull models reconstructed from CT images (black).

Surface deviations between the FE models and post-operative skull models reconstructed from CT images are given in Fig. 6. Surface deviation was relatively low on the frontal and temporal bones, and increased on the posterior skull surfaces expanded by the springs. In particular, the highest values of surface deviations were recorded on the top portion of the posterior flap of Patient 1 and Patient 3. The cross-sections of the FE models simulating spring assisted cranioplasty and post-operative skull growth (orange), and the post-operative CT reconstructions (black) are shown in Fig. 7. The surface deviation of the superior portion of the skull between FE and post-op CT is visible for Patient 1 and slightly less for Patient 3. The FE model and post-operative model of Patient 2 matched fairly well with a slight deviation at the inferior portion of the skull.

The thermal expansion coefficients in the FE models simulating spring assisted correction skull growth between surgical interventions and post-operative CT scans are given in Table 2.

**Table 2:** Thermal expansion coefficients and post-operative ICV in the FE models simulating spring assisted correction and skull growth between surgical interventions and post-operative CT scans.

	Thermal expansion coefficient (K^−1^)	Post-operative ICV from CT measurements (mL)
Patient 1	0.00018	1298
Patient 2	0.00055	957
Patient 3	0.00010	1030

Although a relatively high expansion coefficient was used in the Patient 2 FE model, the increase in the skull size remained relatively small. On the other hand, there was a remarkable increase in the ICV of patient 3 due spring assistance, although a very small thermal expansion coefficient was used in the simulations. The surgery resulted in expansion of the posterior vault of the skull in Patient 1 and Patient 3, whilst for Patient 2, the operation increased mainly the width of the osteotomy rather than the overall skull.

**Figure 8:**
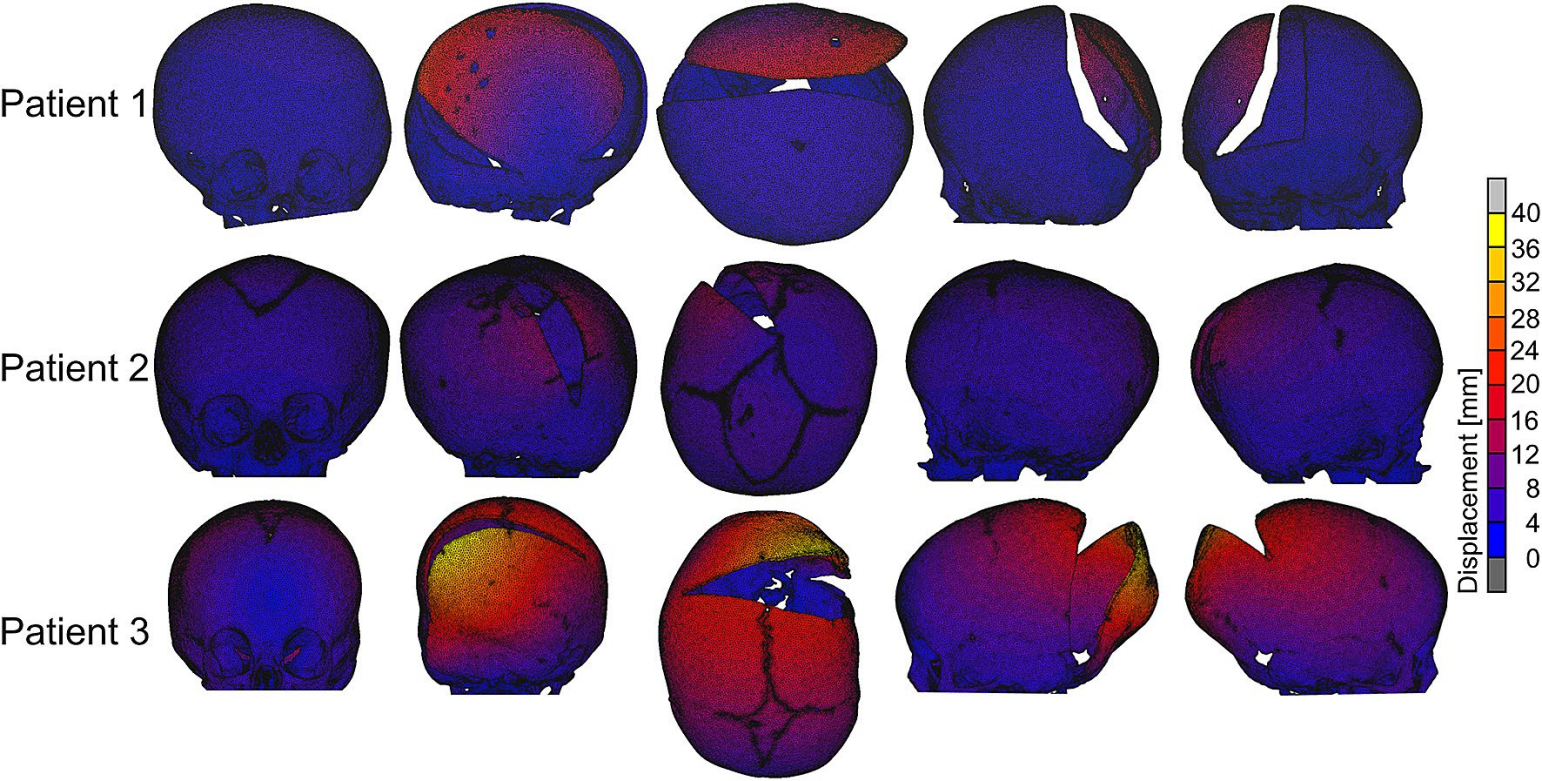
Displacement maps for the skull models for the spring assisted cranioplasty and post-operative skull growth.

Displacement maps for the skull models for the spring assisted cranioplasty and post-operative skull growth are given in Fig. 8. Maximal displacements in the skull models of Patient 1, Patient 2 and Patient 3 were 23.55 mm, 14.33 and 40.01 mm, respectively, with Patient 3 having the largest displacements overall.

## Discussion

In this study, spring assisted cranioplasty was simulated for isolated LC using FE analyses in three different patient specific models. Skull growth between the pre-operative CT imaging and surgical intervention, and after surgical intervention was included in the simulations. The simulation results were validated using post-operative reconstructions from CT images.

Brain growth in infants is driven by biological and genetic mechanisms^41^, and the skull grows in synchrony with the brain^42,43^ through extremely complex signaling pathways and genetic mutations. Interaction between different mechanisms still remains unclear in patients affected by craniosynostosis whereas regulatory mechanisms are extremely complex^44^. Moreover, recent studies suggest that skull growth patterns in craniosynostosis depend on mechanical effects^45^. Therefore, developing a model simulating skull growth remains a challenge. In this study, skull growth was simulated using a relatively simple model similar to thermal expansion, where the amount of skull growth depends on a thermal expansion coefficient, and is driven by a temperature difference, constant across all patient models. The expansion coefficient is tuned for each individual skull FE model: higher expansion coefficients are used when the patient is younger or when there is a longer time span between the pre-operative CT imaging and surgical intervention, or surgical intervention and post-operative CT imaging. The skull growth rate in each patient was personalised through a proportional coefficient (*k*) based on the patient pre-operative ICV and the growth curve developed by Breakey et al.^37^ for healthy children. Relatively high “k” values representing large LC patient intracranial volumes will result in a faster growth rate as “k” is multiplied also with the term including time (Eq. 3) whereas relatively small “k” values will result in a slower growth rate.

The structure of the cranial bones is not homogenous due to very complex developmental mechanisms in the skull^44^ ; therefore, in children, the properties change substantially with age^34^ and are different in the different parts of the skull^34^. In this study, the bones and sutures were assumed homogenous and with the same mechanical behavior for every bony portion as data are not available for the specific patient populations. Although, relatively high elastic modulus values are reported in the literature for cranial bones^34^, the spring assisted cranioplasty FE simulations with selected low values for the material properties, were in good agreement with the post-operative CT scans. Reason for the difference of the material properties could be that the non-homogenous structure of the bones may result in higher elastic modulus values whereas a similar mechanical response from a homogenous material could be obtained with a relatively low elastic modulus value. Nevertheless, it should be noted that the selected values for the material properties are within a biological range for the ages of simulated patients^32–34^.

The simulation results in this study show that the final shape of the skull depends on the performed osteotomies. Relatively longer cuts as performed in Patient 1 and Patient 3 allow mainly hinging and expansion in the cranium whereas a minimal cut as in Patient 2 allows the gap between edges of osteotomy to enlarge. It has already been shown that the size and locations of the osteotomies are crucial for an optimal outcome from surgical operations^31^. The simulation results in this study confirm the findings in the literature.

Although the surface deviation between the FE models and post-operative skull models constructed from CT images remained within a low range, it was relatively high at the back side where the skull was expanded in Patient 1 and Patient 3. Relatively high surface deviations might be because of the complex mechanical properties of cranial bones, such as viscoelasticity, are not included in the simulations.

The spring assisted cranioplasty FE models in this study was simulated by including skull growth and mechanical properties of the bones and sutures (i.e. modulus and passion ratio). Simulating the viscoelastic properties of the cranial bones in the future will allow remodeling of the skull during recovery as a result of mechanical adaptation under spring force^46^. Moreover, properties of bones change over time, therefore, modelling these changes with respect to age rather than modifying only the expansion coefficients for every operation will allow simulating and planning the surgical intervention more accurately. The skull growth in the patient models was evaluated by adapting the healthy subject curve of change of ICV over time (Fig. 2)^37^. Isolated LC is a highly rare syndrome; therefore, a model that can predict the skull growth in these specific patients requires is not yet available. Sutures are the fibrous tissues in between the cranial bones and facilitate the cranial growth^47^. Moreover, they generate bone at edges of the bones by responding the external stimuli^47^. Understanding of this mechanism is still limited^48^; therefore bone formation is not included in the FE models. Nonetheless, it should be noted that despite the limitations, the developed FE models simulated spring assisted cranioplasty in the LC patients accurately.

## Conclusions

The simulation results show the potential of the parametric FE models to simulate surgical outcomes in LC corrected with spring assisted cranioplasty. Replicating spring assisted cranioplasty in LC patients allow tuning of the parameters for surgical planning. Larger studies would allow to determine a population specific set of parameters for these patients in order to use the model prospectively. A parametric study on spring types and locations could then allow optimisation of function and aesthetic outcomes in LC surgical corrections.

## Data Availability

All data generated or analysed during this study are included in this published article.
